# Parent–Child Relationships and Academic Performance of College Students: Chain-Mediating Roles of Gratitude and Psychological Capital

**DOI:** 10.3389/fpsyg.2022.794201

**Published:** 2022-03-31

**Authors:** Jun Li, Jianhao Huang, Ziao Hu, Xiang Zhao

**Affiliations:** ^1^Department of Education Management, Chinese International College, Dhurakij Pundit University, Bangkok, Thailand; ^2^Institute of Digital Economy, School of Economics, Yunnan University, Kunming, China

**Keywords:** gratitude, psychological capital, academic performance, parent–child relationships, college students

## Abstract

This study used the Social Cognitive Theory and Broaden-and-Build Theory to propose and validate a chain mediation model. In total, 417 Chinese college students were studied to explore the effects of parent–child relationships on their academic performance. In addition, we investigated the chain-mediating roles of gratitude and psychological capital. The results showed that (1) the parent–child relationship significantly and positively affected the academic performance of college students; (2) gratitude partially mediated the parent–child relationship and the academic performance of college students; (3) psychological capital partially mediated the parent-child relationship and the academic performance of college students; and (4) gratitude and psychological capital exerted a chain-mediating effect between parent–child relationships and the academic performance of college students. Based on the results of the study, we conclude that the parent–child relationship not only directly affects the academic performance of college students but also indirectly affects it through the chain mediation of gratitude and psychological capital. Moreover, we proposed reasonable suggestions on how colleges and universities can guide students to deal with parent-child relationships, strengthen gratitude education, and improve psychological capital.

## Introduction

Parent–child relationship is a two-way interaction between parents and their children (Robinson, 2015). It is an important environmental factor influencing an individual’s adaptation and development (Miller et al., 2000; Slinner and Steinhauer, 2000; Bronfenbrenner, 2005; Popov and Ilesanmi, 2015; Yang, 2018; Steele and Cliff, 2019). Recent studies have shown that the parent-child relationship has been involved in the learning process of students (Tus, 2021; Zhu et al., 2021), which has emerged as a major factor affecting their academic performance and daily life (Carmona-Halty et al., 2020; Liu et al., 2021).

Data from a follow-up survey in China reported that parent–child relationships significantly affected students’ academic performance (Lu et al., 2021). A recent longitudinal study has reported that good parent–child relationships can reduce the pressure and irritability of teenagers, which is a protective factor for their daily study and life (Janssens et al., 2021), with a positive impact on the academic performance of students during online learning (Tus, 2021). Previous studies have stated that the parent–child relationship is a significant predictor of students’ academic performance (Carmona-Halty et al., 2020). Moreover, a good parent-child relationship promotes academic self-efficacy, thereby reducing academic stress, which is vital for successful academic performance (Mulyadi et al., 2016; Yuan et al., 2016). Accordingly, we suggest that the parent-child relationship is an essential family environment factor directly affecting the academic performance of college students.

Parent–child relationships not only directly affect the academic performance but also an individual’s positive emotions (Dunn and Brown, 1994; Xu et al., 2008; Deng et al., 2009; Obeldobel and Kerns, 2021). Previous research has reported parent-child relationship as a root factor in developing individual gratitude (Obeldobel and Kerns, 2021) that can significantly predict an individual’s gratitude (Wu et al., 2016; Wang and Du, 2019). In addition, the parent–child relationship is an antecedent variable of psychological capital and significantly and positively impacts the individual psychological capital (Kwok et al., 2015; Chen et al., 2017; Nolzen, 2018; Carmona-Halty et al., 2020; Choi, 2021). Recent studies have reported that gratitude and psychological capital allow students to cope positively with their study and life; these are critical for college students (Du and Chen, 2021; Maykrantz et al., 2021; Wang et al., 2021; Zainoodin et al., 2021). Individuals with high gratitude and psychological capital levels have better academic performance (Sevari and Farzadi, 2018; Carmona-Halty et al., 2019; Martínez et al., 2019; Clarkson, 2020; Slåtten et al., 2021; Zainoodin et al., 2021). Furthermore, previous studies have frequently explored gratitude and psychological capital as important mediating variables (Yang, 2018; Carmona-Halty et al., 2020; Ling et al., 2020; Li et al., 2021). Therefore, this study focuses on two critical personal factors—gratitude and psychological capital—to explore their functions in mediating parent–child relationships and the academic performance of college students.

Previous research on parent-child relationships has focused on children and high-school students (Carmona-Halty et al., 2020; Niu et al., 2020; Janssens et al., 2021). However, relatively little research has been conducted on the effects on college students. Because college students still need to maintain close relationships with their families, parent-child relationships are crucial for the academic performance of college students (Fingerman et al., 2016; Steele and Cliff, 2019). Although previous research has reported the relationship between the four variables of parent–child relationships, gratitude, psychological capital, and academic performance, a few discussions have explored the chain-mediating roles of gratitude and psychological capital on the effect of parent–child relationships and the academic performance of college students. Previous research suggested that exploring students’ academic performance should begin with family parent–child relationships (Carmona-Halty et al., 2020). Therefore, we used the Social Cognitive Theory and Broaden-and-Build Theory, and regarded the parent-child relationship as an essential environmental factor, affecting the academic performance of college students. In addition, we regarded gratitude and psychological capital as two key personal factors to explore the chain-mediating effect of gratitude and psychological capital on parent–child relationships and the academic performance of college students.

### Parent–Child Relationships and Academic Performance

Previous research has concentrated on the definition, theoretical construction, influencing factor models, and methods of measuring academic performance. The definition of academic performance has undergone continuous enrichment from traditional test scores to comprehensive performance (Lee and Shute, 2010; Gao and Chen, 2016; Li et al., 2016; Andrés et al., 2017). This study recognized that the academic performance of college students should be comprehensively explored rather than just rating test scores, citing Chinese scholars Wang et al., who reported that academic performance includes learning efficiency, interpersonal promotion, learning dedication, and objective achievement (Wang et al., 2011). Social Cognitive Theory explains the effect of the environment on behavior (Bandura, 1986). In addition, related studies have explored the family as an essential environmental factor affecting an individual’s academic performance, suggesting that a good home environment and family support can enhance academic performance (Shek, 1997; Yuan et al., 2016; Fan, 2019; Du and Zhang, 2020). For example, students who receive adequate care and support at home will have better academic outcomes (Martin and Dowson, 2009; Cheng et al., 2012; Carmona-Halty et al., 2019; Sun et al., 2021).

Previous studies reported that the parent–child relationship serves as a vital family environment factor, influencing an individuals’ academic performance (Yuan et al., 2016; Carmona-Halty et al., 2020). The parent–child relationship is an interpersonal relationship that an individual is initially exposed to Wu (2017), Ling et al. (2018). This relationship synthesizes parenting styles, emotional expressions, and values; in addition, it is an innate environmental factor that influences an individual’s adaptability, mental health, and academic performance (Fang et al., 2004; Popov and Ilesanmi, 2015; Yang, 2018; Steele and Cliff, 2019; Carmona-Halty et al., 2020). Research has reported that good parent–child relationships can satisfy an individual’s basic emotional needs, such as a sense of belonging, leading to positive academic exploration and pursuit (Martin and Dowson, 2009; Carmona-Halty et al., 2020). Effective communication between parents and children reduces negative stress (Janssens et al., 2021; Tang et al., 2021; Zhu et al., 2021) and significantly and positively affects their academic performance (Carmona-Halty et al., 2020; Tus, 2021). Previous research has shown that the parent–child relationship can directly predict a student’s academic performance (Mulyadi et al., 2016; Yuan et al., 2016; Carmona-Halty et al., 2020). In summary, this study states that the parent–child relationship is an important environmental factor that impacts the academic performance of college students and proposed the hypothesis H1: Parent–child relationships significantly and positively influence the academic performance of college students.

### Mediating Role of Gratitude

Gratitude is a positive emotion that refers to a life orientation that focuses on and appreciates positive things (Watkins et al., 2003). In addition, it promotes individuals to develop a positive perception and feedback behavior for external assistance (Wood et al., 2010; Obeldobel and Kerns, 2021). Studies have reported that among the several factors triggering emotional problems in adolescents, the family aspect is the primary factor (Xu et al., 2008). A literature review of gratitude and parent–child relationships states that the parent–child relationship is the root of gratitude, an essential context for developing gratitude, and a significant factor influencing individual gratitude (Obeldobel and Kerns, 2021). Previous research indicates that healthy parent-child relationships have an irreplaceable function in perceiving, expressing, and cultivating positive emotions (Dunn and Brown, 1994; Deng et al., 2009). Good parent–child relationships are conducive to stimulating an individual’s gratitude (Niu et al., 2020). In addition, it can significantly and positively predict individual gratitude (Wu et al., 2016; Wang and Du, 2019; Ling et al., 2020). Thus, this study suggests that parent-child relationships significantly and positively impact the gratitude in college students.

The concept of gratitude states that it is not only a positive life orientation but also tends to promote individuals’ motivation and behavior to generate feedback (Wood et al., 2010; Obeldobel and Kerns, 2021). Research has reported gratitude as an essential personal factor in assisting college students to cope with study and life more positively (Zainoodin et al., 2021). Gratitude motivates individuals to continuously improve themselves and make progress (Armenta et al., 2017). Furthermore, individuals with a high level of gratitude have a higher sense of giving back and more precise goals in life and learning, thus motivating them to pursue their academic goals with gratitude, persevere, and achieve more satisfactory academic performance (Bono and Froh, 2009; Yu et al., 2011; Berlanga-Silvente et al., 2017). In addition, previous studies have confirmed that gratitude promotes students’ academic engagement and thus academic output (Wen et al., 2010; Yu, 2015). This is a significant predictor of academic performance in college students (Sevari and Farzadi, 2018; Clarkson, 2020; Zainoodin et al., 2021). According to the Social Cognitive Theory, environmental factors influence behavior by affecting personal factors (Bandura, 1986). Research has reported that parent-child relationships directly affect academic performance and can indirectly affect academic performance by influencing an individual’s gratitude (Li et al., 2021). In conclusion, this study suggests that parent-child relationships influence academic performance by influencing college students’ gratitude, and proposes hypothesis H2: Gratitude plays a mediating role between parent-child relationships and college students’ academic performance.

### Mediating Role of Psychological Capital

Psychological capital was first introduced by the American economist, Goldsmith who considered it to be consisting of several psychological characteristics that can improve an individual’s productivity and performance (Goldsmith et al., 1998). This concept has rapidly and significantly impacted economics, management, and educational psychology (Li et al., 2014; Nolzen, 2018; Choi, 2021; Effendi et al., 2021). Increasing research in recent years has identified several family environmental factors (e.g., parent–child relationships, family support, etc.) as antecedent variables of psychological capital with significant predictive effects on psychological capital (Kwok et al., 2015; Chen et al., 2017; Carmona-Halty et al., 2020; Choi, 2021). Families provide the necessary environmental support to develop high levels of psychological capital. In addition, individuals with a high-quality parent–child relationship have higher levels of psychological capital (Kwok et al., 2015; Carmona-Halty et al., 2020). Good parent-child communication and exchange can positively predict an individual’s psychological capital (Choi, 2021). Therefore, this study suggests that parent–child relationships significantly and positively affect the psychological capital of college students.

Several studies have reported that psychological capital is a crucial personal factor to secure the academic life of college students (Du and Chen, 2021; Huang and Zhang, 2021; Maykrantz et al., 2021; Wang et al., 2021). Psychological capital serves as a deep-seated motivator, leading the students to positive learning behaviors and ensuring efficient learning outcomes (Luthans, 2002; Luthans et al., 2007). Research has confirmed that psychological capital promotes academic engagement, thereby reducing academic burnout in college students (Wang et al., 2021). Psychological capital levels correlate with academic performance; furthermore, high levels of psychological capital are predictors of successful academic performance (Carmona-Halty et al., 2019; Martínez et al., 2019; Slåtten et al., 2021). The Social Cognitive Theory suggests that the environment affects an individual’s psychological factors and consequently an individual’s behavior (Bandura, 1986). Previous research has reported that psychological capital can explain the intrinsic association between parent–child relationships and academic performance (Carmona-Halty et al., 2020). Good parent–child relationships can satisfy an individual’s basic emotional needs and prompt psychological capital such as optimism that motivates an individual toward positive academic exploration and successful academic performance (Martin and Dowson, 2009; Carmona-Halty et al., 2019). Therefore, this study suggests that parent–child relationships affect academic performance by influencing college students’ psychological capital. Therefore, we propose the following hypothesis H3: Psychological capital plays a mediating role between parent-child relationships and college students’ academic performance.

### Parent–Child Relationships, Gratitude, Psychological Capital, and Academic Performance

Both gratitude and psychological capital are important predictors of students’ academic performance and act as a mediator between parent-child relationships and academic performance (Carmona-Halty et al., 2020; Li et al., 2021). However, only a few studies have directly focused on the relationship between gratitude and psychological capital. For example, previous research reported gratitude as a broader life orientation at the personality level, one that is significantly related to and differentiated from the core elements of psychological capital, such as optimism, hope, and resilience (McCullough et al., 2002, 2004; Wood et al., 2010; Smith et al., 2014; Shiota et al., 2017; Obeldobel and Kerns, 2021). However, studies have not explored the relationship between the effects of gratitude on psychological capital. According to the Broaden-and-Build Theory, gratitude is one of the most representative positive emotions that can expand an individual’s thinking and cognitive scope; thus, constructing lasting psychological resources (e.g., resilience, optimism, etc.) (Fredrickson, 2004, 2013). Research has demonstrated gratitude as a significant positive predictor of psychological capital (Liao, 2016). In addition, psychological capital mediates the relationship between gratitude and learning burnout (Fu, 2021). Therefore, this study suggests that college students with higher levels of gratitude have correspondingly higher levels of psychological capital and propose the following hypothesis H4: Gratitude has a significantly positive predictive effect on psychological capital.

The Social Cognitive Theory explains the interaction between environment, individual, and behavior (Bandura, 1986). Previous research has demonstrated that parent-child relationships affect academic performance by influencing gratitude (Li et al., 2021) and psychological capital (Carmona-Halty et al., 2020). Based on the Broaden-and-Build Theory and empirical studies, gratitude significantly predicts psychological capital (Fredrickson, 2004, 2013; Fu, 2021). Therefore, this study speculated that the parent–child relationship affects the academic performance of college students through chain mediation of gratitude and psychological capital. We propose the following hypothesis H5: Gratitude and psychological capital exert a chain-mediating effect between parent–child relationships and academic performance of college students.

**FIGURE 1:**
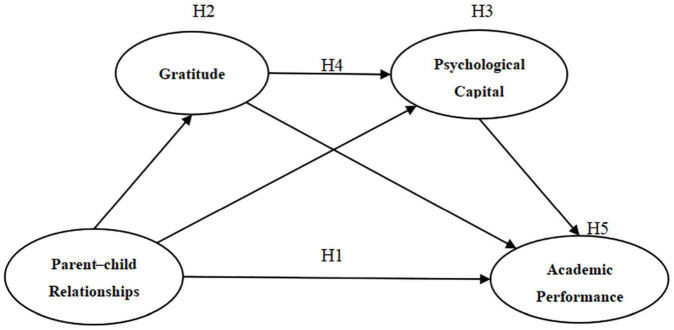
Hypothetical model.

In summary, the hypothetical model for this study is shown in Figure 1.

## Research Method

### Participants and Sampling Procedure

We made use of an online questionnaire. Data were collected between April 21 and April 30, 2021. Before the questionnaires were filled out and submitted on the spot, the head teacher explained the purpose of the research to the participants and obtained their consent. A total of 465 questionnaires were distributed to four colleges and universities in the Yunnan Province, with 417 valid questionnaires remaining after excluding invalid questionnaires with a short response time and missing answers, yielding a 90% valid response rate. The respondents included 153 (36.7%) males and 264 (63.3%) females; 132 (31.7%) first-year students, 93 (22.3%) second-year students, 161 (38.6%) third-year students, and 31 (7.4%) fourth-year students; 223 (53.5%) from humanities and social science-related majors, 151 (36.2%) from science and engineering-related majors, and 43 (10.3%) from agricultural and medicine-related majors. Furthermore, 203 (48.7%) students had families living in cities, 76 (18.2%) in townships, and 138 (33.1%) in rural areas. In addition, 176 (42.2%) students were only children, whereas 241 (57.8%) had siblings. The age of students in the first to the fourth year of college ranged from 18 to 23 years.

### Measures

SPSS 21.0 was used to compile the sample descriptive statistics, examine the reliability of the questionnaire, and perform correlation analysis. AMOS 21.0 was used to conduct Confirmatory Factor Analysis (CFA) and Common Method Variance (CMV), construct Structural Equation Model (SEM), and validate them with the non-parametric percentile bootstrap method.

#### Parent–Child Relationships

We used the questionnaire of college students’ parent-child interaction to measure their parent-child relationships (Wang et al., 2017). The questionnaire, with 21 questions, has four dimensions: active care (e.g., I will take the initiative to ask my parents about their health.), self-disclosure (e.g., I will communicate with my parents about trivial things in life.), family activities (e.g., my family likes to have family dinners.), economic communication (e.g., I will take the initiative to report my income and expenses to my parents.). It is a 5-point Likert scale ranging from 1 (totally inconsistent) to 5 (totally compliant), with higher scores indicating better parent-child relationships. Table 1 shows the results of the Confirmatory Factor Analysis (CFA), with the Standardized Factor Loading (SFL) ranging from 0.56 to 0.88, all of which were greater than 0.5, showing good validity of the scale (Hair et al., 1992). Composite Reliability (CR) values ranged from 0.74 to 0.94, greater than 0.6; and the values of Average Variance Extracted (AVE) ranged from 0.43 to 0.72, greater than 0.4. The results indicated that the scale has convergent validity (Fornell and Larcker, 1981). The model fit index was *x^2^/df* = 4.22, GFI = 0.83, NFI = 0.90, TLI = 0.90, CFI = 0.91, PNFI = 0.78, indicating that the measurement model fit was acceptable (McDonald and Ho, 2002; Hsiao et al., 2016). Cronbach’s α was 0.94 for the active care dimension, 0.94 for the self-disclosure dimension, 0.86 for the family activities dimension, and 0.74 for the economic communication dimension. Cronbach’s α values for all four dimensions were greater than 0.7, indicating good reliability of the scale (Nunnally, 1994).

**TABLE 1:** Confirmatory factor analysis of the questionnaire of college students’ parent–child interaction.

Dimension	Item	SFL	CR	AVE
Active care	(1) I will take the initiative to ask my parents about their health.	0.87	0.94	0.71
	(2) I will take the initiative to ask my parents about their work, and I will comfort them when they are in a bad mood.	0.87		
	(3) I will take the initiative to praise and compliment my parents.	0.85		
	(4) I will communicate with my parents about their past events.	0.84		
	(5) I will teach my parents about high-technology products and the current popular culture.	0.84		
	(6) I care about my parent’s health, and if they are sick, I will go home to visit them.	0.79		
Self-disclosure	(1) I usually feel good after communicating with my parents.	0.87	0.94	0.72
	(2) I will communicate with my parents about trivial things in life.	0.88		
	(3) After communicating with my parents, I usually feel that the communication went well.	0.85		
	(4) I communicate with my parents about my interpersonal relationships.	0.85		
	(5) I will communicate with my parents about my hobbies and interests.	0.86		
	(6) I will communicate with my parents about my study.	0.77		
Family activities	(1) I will go on trips with my family.	0.76	0.86	0.56
	(2) My family gives gifts to each other.	0.71		
	(3) My family likes to have family dinners.	0.73		
	(4) I have a common hobby with other family members.	0.79		
	(5) I communicate with my parents weekly (including phone calls, WeChat, going home).	0.75		
Economic communication	(1) I will take the initiative to ask my parents to buy things for me (clothes, cell phone, computer, etc.).	0.75	0.74	0.43
	(2) I will get angry or argue with my parents when they cannot fulfill my wishes.	0.61		
	(3) I will take the initiative to ask my parents for living expenses.	0.67		
	(4) I will take the initiative to report my income and expenses to my parents.	0.56		

*SFL, standardized factor loading; CR, composite reliability; AVE, average variance extracted.*

#### Academic Performance

We used the College Students’ Academic Performance scale (Wang et al., 2011), which has 19 questions and the following four dimensions: learning efficiency (e.g., the extent to which I complete my study tasks within the specified time), interpersonal promotion (e.g., the extent to which I interact with my classmates), learning dedication (e.g., the extent of my initiative in seeking challenging learning tasks), and objective achievement (e.g., my intellectual performance compared to the class average.). It is a 5-point Likert scale ranging from 1 (not at all able or lowest) to 5 (fully able or highest), with higher scores indicating higher academic performance. Table 2 shows the results of the CFA, with the SFL ranging from 0.73 to 0.88, greater than 0.5, indicating good validity of the scale. The values of CR ranged from 0.87 to 0.92, greater than 0.6; and the values of AVE ranged from 0.65 to 0.70, greater than 0.4. The results indicated that the scale has convergent validity. The model fit index was *x^2^/df* = 3.69, GFI = 0.87, NFI = 0.92, TLI = 0.93, CFI = 0.94, PNFI = 0.78, indicating that the measurement model fit was satisfactory. Cronbach’s α for the learning efficiency dimension of the scale was 0.91, 0.92 for the interpersonal promotion dimension, 0.87 for the learning dedication dimension, and 0.88 for the objective achievement dimension. Cronbach’s α values for all four dimensions were greater than 0.7, indicating good reliability of the scale.

**TABLE 2:** Confirmatory factor analysis of the college students’ academic performance scale.

Dimension	Item	SFL	CR	AVE
Learning efficiency	(1) The extent to which I complete my study tasks within the specified time.	0.79	0.92	0.65
	(2) The extent to which I complete my work tasks as per teacher’s requirements.	0.84		
	(3) The extent to which I complete my assignments on time.	0.82		
	(4) The extent to which my learning results meet the expected goals.	0.81		
	(5) The extent to which I strictly follow school rules and regulations.	0.73		
	(6) The quality of my study.	0.83		
Interpersonal promotion	(1) The extent to which I am considerate and caring to other students	0.83	0.92	0.66
	(2) The extent to which I take the initiative to provide help to other students	0.85		
	(3) The extent to which I interact with my classmates	0.83		
	(4) The extent to which I am fair to my classmates.	0.83		
	(5) The extent of my enthusiasm in participating in team activities.	0.73		
	(6) The extent to which I cooperate with other students in my studies.	0.81		
Learning dedication	(1) The extent of my initiative in seeking challenging learning tasks.	0.79	0.87	0.70
	(2) The extent of my proactive approach to solving problems in learning.	0.84		
	(3) The extent of my persistence in overcoming difficulties to complete learning tasks.	0.88		
Objective achievement	(1) My overall performance compared to the class average.	0.87	0.88	0.66
	(2) My intellectual performance compared to the class average.	0.78		
	(3) My moral performance compared to the class average.	0.82		
	(4) My cultural and sports performance compared to the class average.	0.76		

*SFL, standardized factor loading; CR, composite reliability; AVE, average variance extracted.*

#### Gratitude

The Adolescent Gratitude Scale (AGS) was used to assess college students’ levels of gratitude (He et al., 2012). The scale consists of 23 questions and the following six dimensions: perception and experience of social favors (e.g., I frequently feel happy to live in today’s society), expressing and reciprocating to nature favors (e.g., I am willing to contribute to the greening and beautification of nature), perception and experience of natural favors (e.g., I am often absorbed in the beauty of nature), expressing and reciprocating to others favors (e.g., I always express my gratitude to those who care and help me in time), expressing and reciprocating to social favors (e.g., I will do some good deeds to repay society when I have the opportunity), perception and experience of others favor (e.g., people who help me want something from me, so I do not need to be grateful to others for their help; this dimension is reversed). It is a 5-point Likert scale, ranging from 1 (totally inconsistent) to 5 (totally compliant), with higher scores indicating higher levels of gratitude. The questions from the sixth dimension of this scale are reverse questions; data analysis was conducted after removing it (Kline, 2010). Table 3 shows the results of CFA after removing the sixth dimension. SFL ranged from 0.77 to 0.93, all greater than 0.5, indicating good validity of the scale. The CR values ranged from 0.90 to 0.95, all greater than 0.6, and the AVE values ranged from 0.72 to 0.83, all greater than 0.4, indicating that the scale has convergent validity. The model fit index was *x^2^/df* = 4.21, GFI = 0.86, NFI = 0.93, TLI = 0.94, CFI = 0.95, PNFI = 0.79, indicating that the fitness of the measurement model is acceptable. Cronbach’s α was 0.93 for the dimension of perception and experience of social favors, 0.95 for the dimension of expressing and reciprocating from natural favors, 0.92 for the dimension of perception and experience of natural favors, 0.95 for the dimension of expressing and reciprocating from others favors, and 0.90 for expressing and reciprocating from social favors. The values of Cronbach’s α for all dimensions were greater than 0.7, indicating good reliability of the scale.

**TABLE 3:** Confirmatory factor analysis of the Adolescent Gratitude Scale (AGS).

Dimension	Item	SFL	CR	AVE
Perception and experience of social favors	(1) I frequently feel happy to live in today’s society	0.77	0.93	0.72
	(2) I am grateful that I was born in this great nation and country	0.89		
	(3) My country and society have provided good conditions for my growth.	0.93		
	(4) School has taught me many wonderful things	0.86		
	(5) The community makes my life full of joy and warmth.	0.77		
Expressing and reciprocating from natural favors	(1) I care for public property.	0.88	0.95	0.83
	(2) I protect nature by doing small things around me.	0.92		
	(3) I care for every plant and tree in nature.	0.93		
	(4) I am willing to contribute to the greening and beautification of nature.	0.91		
Perception and experience of natural favors	(1) I am grateful for the beautiful feeling I get when I notice things around me.	0.93	0.92	0.74
	(2) I am often absorbed in the beauty of nature.	0.89		
	(3) I feel that the most beautiful music is the heavenly music of nature.	0.80		
	(4) I believe that the sun, air, wind, frost, snow, and rain are all gifts from nature to us.	0.82		
Expressing and reciprocating from others favors	(1) I remember all my friends’ care and contribution to me.	0.91	0.95	0.81
	(2) The care and help of others often touch me.	0.87		
	(3) I always express my gratitude to those who care and help me in time.	0.92		
	(4) I do my best to help my friends who have helped me.	0.91		
Expressing and reciprocating from social favors	(1) I will do some good deeds to repay society when I have the opportunity.	0.90	0.90	0.74
	(2) I am enthusiastic about social welfare and often take part in public welfare activities.	0.83		
	(3) I always repay my teachers for their teaching and care in some way.	0.86		

*SFL, standardized factor loading; CR, composite reliability; AVE, average variance extracted.*

#### Psychological Capital

We used the psychological capital scale in this study (Wang et al., 2011), which has four dimensions, totaling 15 questions: self-confidence (e.g., I can present my ideas confidently at team events or meetings), tenacity (e.g., I can always recover quickly from setbacks), optimism (e.g., When I encounter difficulties, I always believe that “the sun always shines after the storm”), and responsibility (e.g., I will do my best to fulfill the responsibilities I have undertaken). It is a 5-point Likert scale, ranging from 1 (totally inconsistent) to 5 (totally compliant), with higher scores indicating higher levels of psychological capital. Table 4 shows the results of CFA, with SFL ranging from 0.53 to 0.93, greater than 0.5, indicating good validity of the scale. The values of CR ranged from 0.77 to 0.94, greater than 0.6, and the values of AVE ranged from 0.46 to 0.79, greater than 0.4, indicating the scale has convergent validity. The model fit index was: *x^2^/df* = 4.20, GFI = 0.89, NFI = 0.92, TLI = 0.93, CFI = 0.94, PNFI = 0.74, indicating that the fitness of the measurement model is acceptable. Cronbach’s α was 0.85 for the dimension of self-confidence, 0.89 for the dimension of tenacity, 0.77 for optimism, and 0.94 for responsibility. The values of Cronbach’s α for all dimensions were greater than 0.7, indicating good reliability of the scale.

**TABLE 4:** Confirmatory factor analysis of the psychological capital scale.

Dimension	Item	SFL	CR	AVE
Self-confidence	(1) I can present my ideas confidently at team events or meetings.	0.78	0.84	0.64
	(2) I enjoy challenging work assignments.	0.77		
	(3) I believe I can play a role in team activities.	0.85		
Tenacity	(1) I can always handle pressure with ease.	0.85	0.89	0.67
	(2) I do not get discouraged even if my work and studies are not going well.	0.83		
	(3) I can always recover quickly from setbacks.	0.84		
	(4) I can cope with unexpected situations when I am depressed.	0.75		
Optimism	(1) When I encounter something uncertain, I usually expect the best outcome.	0.58	0.77	0.46
	(2) When I encounter difficulties, I always believe that “the sun always shines after the storm.”	0.80		
	(3) Things always turn out the way I want them to.	0.53		
	(4) I believe that “you reap what you sow.”	0.76		
Responsibility	(1) I will do my best to fulfill the responsibilities I have undertaken.	0.90	0.94	0.79
	(2) I will try to do what I have promised.	0.93		
	(3) I will never pass on my share of responsibility to others.	0.85		
	(4) Even if there are difficulties in completing the task, I will persevere responsibly to the end.	0.87		

*SFL, standardized factor loading; CR, composite reliability; AVE, average variance extracted.*

#### Common Method Variance Test

Confirmatory factor analysis was used to test the problem of CMV. Table 5 shows the results of comparing the CFA fit between the multi-factor model and the single-factor model. The chi-square value of the multi-factor model (*x^2^* = 5924.35) was considerably lower than that of the single-factor model (*x^2^* = 14937.39), indicating the multi-factor model fitted significantly better than the single-factor model (Δ*x^2^* = 9013.04, Δ*df* = 136, *p* < 0.001) and no serious CMV problem existed in the study (Verhagen and Van Dolen, 2011).

**TABLE 5:** Comparison of multi-factor and single-factor models.

Model	*x^2^*	*df*	*x^2^/df*	△*x*^2^	△*df*	*p*
Single-factor model	14937.39	2700	5.53	9013.04	136	0.000
Multi-factor model	5924.35	2564	2.31			

## Results

### Descriptive Statistics and Correlation Analysis

Table 6 illustrates the results of descriptive statistics. The current status of each variable is from the medium to a high level. Correlation analysis revealed significant correlations among the variables (Cohen et al., 2009). Because certain dimensions have high correlations, we adopted the discriminant validity test; the results are shown in Table 6. The square root of the AVE of each dimension was greater than the correlation coefficient of each dimension, accounting for more than 75%. It met the criteria for assessing discriminant validity (Fornell and Larcker, 1981), indicating that all variables in this study had good discriminant validity.

**TABLE 6:** Descriptive statistics, correlation analysis and discriminant validity.

Dimension	1	2	3	4	5	6	7	8	9	10	11	12	13	14	15	16	17
PCR1	***0.84***																
PCR2	0.85	***0.85***															
PCR3	0.75	0.77	***0.75***														
PCR4	0.40	0.45	0.52	***0.66***													
GR1	0.66	0.67	0.58	0.29	***0.85***												
GR2	0.65	0.63	0.56	0.22	0.82	***0.91***											
GR3	0.59	0.58	0.49	0.21	0.79	0.85	***0.86***										
GR4	0.60	0.58	0.49	0.21	0.78	0.85	0.87	***0.90***									
GR5	0.59	0.57	0.54	0.28	0.66	0.71	0.75	0.79	***0.86***								
PC1	0.47	0.45	0.39	0.21	0.41	0.45	0.43	0.45	0.45	***0.80***							
PC2	0.55	0.53	0.43	0.26	0.46	0.47	0.44	0.46	0.45	0.71	***0.82***						
PC3	0.53	0.54	0.48	0.33	0.50	0.48	0.44	0.46	0.45	0.63	0.69	***0.68***					
PC4	0.57	0.52	0.39	0.17	0.58	0.64	0.61	0.67	0.58	0.63	0.60	0.67	***0.89***				
AP1	0.60	0.57	0.48	0.31	0.55	0.63	0.59	0.64	0.58	0.61	0.60	0.65	0.79	***0.81***			
AP2	0.71	0.67	0.59	0.39	0.66	0.67	0.62	0.69	0.64	0.59	0.58	0.65	0.74	0.80	***0.81***		
AP3	0.63	0.59	0.52	0.39	0.48	0.53	0.50	0.51	0.55	0.59	0.57	0.59	0.61	0.74	0.74	***0.84***	
AP4	0.64	0.61	0.54	0.33	0.52	0.56	0.51	0.56	0.54	0.52	0.51	0.55	0.58	0.71	0.70	0.70	***0.81***
*M*	3.68	3.69	3.43	2.99	3.92	4.09	4.02	4.12	3.75	3.29	3.28	3.36	3.83	3.62	3.68	3.44	3.53
*SD*	0.95	0.97	0.92	0.85	0.96	0.96	0.94	0.95	1.00	0.99	0.95	0.87	1.00	0.86	0.85	0.89	0.86

*The bold and italic numbers in the diagonal are the square root of AVE. Numbers in the lower diagonal denote the correlation coefficients of two dimensions. The correlation coefficients all reached significant levels (p < 0.001). M, mean; SD, standard deviation; AVE, average variance extracted; PCR, parent–child relationships; AP, academic performance; GR, gratitude; PC, psychological capital; PCR1, active care; PCR2, self-disclosure; PCR3, family activities; PCR4, economic communication; GR1, perception and experience of social favors; GR2, expressing and reciprocating from natural favors; GR3, perception and experience of natural favors; GR4, expressing and reciprocating from others favors; GR5, expressing and reciprocating from social favors; PC1, self-confidence; PC2, tenacity; PC3, optimism; PC4, responsibility; AP1, learning efficiency; AP2, interpersonal promotion; AP3, learning dedication; AP4, objective achievement.*

### Parent–Child Relationships and Academic Performance of College Students

**FIGURE 2:**
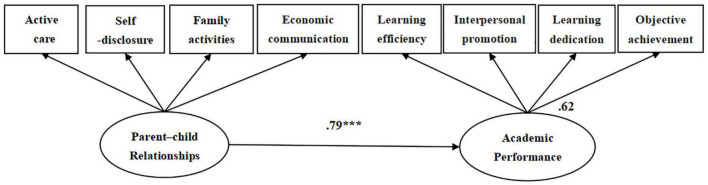
Main effects of parent–child relationships on the academic performance. ***p < 0.001.

Structural Equation Model (SEM) was used to construct the main effect model of the parent-child relationship on academic performance (Figure 2). The model fit index was as follows: *x^2^* = 92.53, *df* = 19, RMR = 0.03, GFI = 0.95, CFI = 0.97, NFI = 0.97, TLI = 0.96, PNFI = 0.66. The results showed that parent–child relationships can significantly and positively predict the academic performance of college students (β = 0.79, *p* < 0.001), and parent-child relationships can explain 62% of academic performance (SMC = 0.62).

### Chain-Mediating Effect of Gratitude and Psychological Capital Between the Parent-Child Relationship and Academic Performance

**FIGURE 3:**
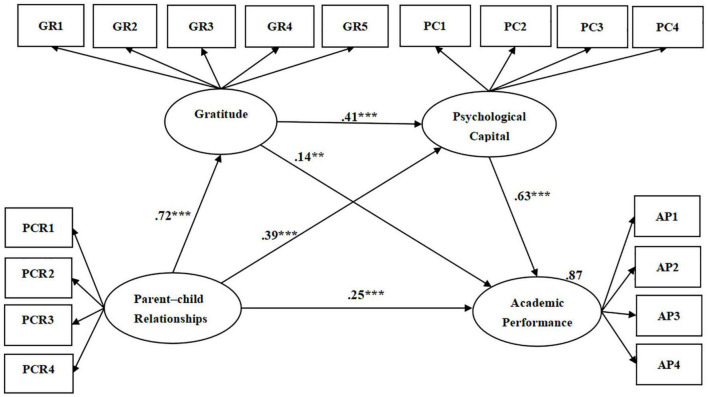
Structural equation model of the influence of parent–child relationships, gratitude, and psychological capital on academic performance. PCR, parent-child relationships; AP, academic performance; GR, gratitude; PC, psychological capital; PCR1, active care; PCR2, self-disclosure; PCR3, family activities; PCR4, economic communication; GR1, perception and experience of social favors; GR2, expressing and reciprocating from natural favors; GR3, perception and experience of natural favors; GR4, expressing and reciprocating from others favors; GR5, expressing and reciprocating from social favors; PC1, self-confidence; PC2, tenacity; PC3, optimism; PC4, responsibility; AP1, learning efficiency; AP2, interpersonal promotion; AP3, learning dedication; AP4, objective achievement. **p < 0.01 ***p < 0.001.

SEM was used to construct the chain-mediating model (Figure 3), and the model fit index was as follows: *x^2^* = 565.26, *df* = 114, RMR = 0.04, GFI = 0.85, CFI = 0.93, NFI = 0.92, TLI = 0.92, PNFI = 0.76, indicating that the fit of chain-mediating model was ideal. To illustrate that the fit of the chain-mediating model has significant difference from other models, this study used SEM to construct the following four models: Model 1 (PCR→AP), model 2 (PCR→GR→AP), model 3 (PCR→PC→AP), and model 4 (PCR→GR→PC→AP). The fitness of model 4 was compared with that of the other models. The results showed that model 4 was significantly different from Model 1(△*x^2^* = 472.73, △*df* = 95, *p* < 0.001), significantly different from Model 2 (△*x^2^* = 259.46, △*df* = 52, *p* < 0.001), significantly different from Model 3 (△*x^2^* = 249.63, △*df* = 63, *p* < 0.001). The results indicate that the fitness of the chain-mediating model finally constructed is significantly different from that of other models.

Furthermore, in model 4, the three variables of parent-child relationships, gratitude, and psychological capital in total explained 87% of academic performance (SMC = 0.87). By comparison, the single variable of the parent–child relationship explained 62% of academic performance in model 1 (SMC = 0.62); the two variables of the parent-child relationship and gratitude explained 70% of academic performance in model 2 (SMC = 0.70); the two variables of the parent-child relationship and psychological capital explained 85% of academic performance in model 3 (SMC = 0.85). The comparison indicates that the chain-mediating of model 4 has the highest explanatory power and can better explain the variance in the academic performance of college students.

#### Direct Effect Analysis

Figure 3 shows that parent-child relationships exerted a significant direct effect on academic performance (β = 0.25, *p* < 0.001). The 95% confidence interval (CI) of the direct effect of parent-child relationships on the academic performance measured by the bias-corrected percentile bootstrap method was 0.125–0.371, excluding 0 (Table 7), validating the significance of the direct effect after gratitude and psychological capital were added as mediating variables; thus, H1 is valid. However, the path coefficient was reduced from 0.79 (*p* < 0.001) in the main effects model to 0.25 (*p* < 0.001) in the chain-mediating model, indicating that gratitude and psychological capital partially mediated the effects of parent-child relationships on academic performance.

**TABLE 7:** Results of the bootstrap analysis.

Effect	Path	Estimate	95% LLCI	95% ULCI
Direct effect	PCR → AP GR → PC	0.25*** 0.41***	0.125 0.266	0.371 0.544
Indirect effect Total indirect effect	PCR → GR→ AP PCR → PC→ AP PCR → GR → PC → AP PCR → AP	0.10** 0.25** 0.19** 0.54**	0.025 0.135 0.099 0.450	0.168 0.308 0.242 0.631
Total effect	PCR → AP	0.79**	0.697	0.844

**p < 0.01, ***p < 0.001. PCR, parent–child relationships; AP, academic performance; GR, gratitude; PC, psychological capital. LLCI, lower limit of confidence interval; ULCI, upper limit of confidence interval.

Gratitude exerted a significant positive direct effect on psychological capital (β = 0.41, *p* < 0.001). For the direct effect of gratitude on psychological capital measured by the bias-corrected percentile bootstrap method, the 95% CI was 0.266–0.544, excluding 0 (Table 7), indicating a direct effect and supporting H4. This result formed the basis for constructing a chain-mediating model.

Parent–child relationships exerted a significant positive direct effect on gratitude (β = 0.72, *p* < 0.001); gratitude had a significant positive direct effect on academic performance (β = 0.14, *p* < 0.01); parent–child relationships had a significant positive direct effect on psychological capital (β = 0.39, *p* < 0.001); and psychological capital had a significant positive direct effect on academic performance (β = 0.63, *p* < 0.001).

#### Indirect Effect Analysis

The chain-mediating model (Figure 3) consisted of three paths that contributed to the indirect effects of parent–child relationships on academic performance: First, the indirect effect of parent–child relationships on academic performance through gratitude exhibited an estimated value of 0.10 (β = 0.10, *p* < 0.01), and the 95% CI was 0.025–0.168, excluding 0 (Table 7), indicating a significant mediating effect of gratitude in this first path and supporting H2.

Second, the indirect effect of parent-child relationships on academic performance through psychological capital had an estimated value was 0.25 (β = 0.25, *p* < 0.01). The 95% CI was 0.135–0.308, excluding 0 (Table 7), suggesting a significant mediating effect of psychological capital in this second path and supporting H3.

Third, the indirect effect of parent–child relationships on academic performance through gratitude and psychological capital had an estimated value of 0.19 (β = 0.19, *p* < 0.01). The 95% confidence interval was 0.099–0.242, excluding 0 (Table 7), indicating a significant chain-mediating effect of gratitude and psychological capital in this third path and supporting H5.

#### Total Effect Analysis

According to the above analysis, the total indirect effect of parent–child relationships on academic performance was the sum of the indirect effects of three paths, with an estimated value of 0.54 (*p* < 0.01). The 95% CI was 0.450–0.631, excluding 0, again indicating a mediating effect. The estimate of the total effect of the model was 0.79 (*p* < 0.01); the 95% confidence interval was 0.697–0.844, excluding 0 (Table 7), supporting the partially chain-mediating effect hypothesis again.

## Discussion

Based on the results of this study, we report that the parent–child relationship not only had a significant direct effect on the academic performance of college students and indirectly affected it through the chain-mediating function of gratitude and psychological capital. The explanatory power of the chain-mediating model was substantially enhanced, indicating that the three variables of the parent–child relationship, gratitude, and psychological capital comprehensively explained the academic performance of college students.

The results of the study verified H1 and confirmed that the parent-child relationship significantly and positively affected the academic performance of college students, which is consistent with the findings of previous studies (Yuan et al., 2016; Carmona-Halty et al., 2020; Lu et al., 2021), indicating that parent–child relationship is an important family environment factor that influences the academic performance of college students. The findings support the social cognitive theory that suggests that the environment significantly influences behavior (Bandura, 1986). In addition, this result suggests that good parent–child relationship is a protective factor in college students’ daily learning and life, which can reduce academic stress and provide the necessary support in the family environment to ensure high academic performance.

The results verified H2 that gratitude exerted a partially mediating role between parent-child relationships and the academic performance of college students, consistent with the results of previous studies (Li et al., 2021; Obeldobel and Kerns, 2021; Zainoodin et al., 2021). It suggests that a positive parent–child relationship is an essential context for developing individual gratitude, stimulating positive life orientations in college students and promoting their focus on the good things in life, thus generating positive motivation and ultimately resulting in good academic performance. The finding supports the social cognitive theory, which explains that the environment influences the behavior by affecting the individuals (Bandura, 1986). The results suggest parent-child relationship as an essential environmental factor in fostering gratitude to promote academic output among college students. Moreover, gratitude as a personal factor and positive emotion help college students cope with study and life positively (Zainoodin et al., 2021).

The study results verified H3 that psychological capital exerted a partially mediating role between parent–child relationships and the academic performance of college students, which is consistent with the results of previous studies (Martínez et al., 2019; Carmona-Halty et al., 2020). It suggests that healthy parent–child relationships develop an individual’s psychological capital, such as optimism and self-confidence, which sustain individuals’ motivation to learn. College students with high levels of psychological capital have been reported to have high levels of academic performance. The findings again support the social cognitive theory and validate that parent–child relationship is a crucial environmental factor contributing to the development of an individual’s psychological capital—a critical personal factor in ensuring high academic performance among college students (Huang and Zhang, 2021; Maykrantz et al., 2021; Wang et al., 2021).

The study results confirmed H4 that gratitude significantly and positively predicted college students’ psychological capital. This is a critical finding, indicating that people with higher levels of gratitude awareness are more optimistic and confident, and have higher levels of psychological resilience and responsibility than people with lower levels of gratitude awareness. The results support the Broaden-and-Build Theory that suggests that gratitude, as a positive personal emotion, can construct more lasting psychological resources (Fredrickson, 2004, 2013). In addition, this study remedies the lack of past research, examining the relationship between gratitude and the impact of psychological capital. The results suggest that gratitude is a predictor of psychological capital; college students with higher levels of gratitude have correspondingly higher levels of psychological capital than those without it.

The study results verified H5 that gratitude and psychological capital had a chain-mediating effect between parent–child relationships and the academic performance of college students, and this result is also an essential contribution of this study. The findings indicate that the parent–child relationship not only exerts a significant direct effect on college students’ academic performance and indirectly affects the academic performance by promoting college students’ gratitude and thus improving their psychological capital level. The chain relationship of the parent–child relationship, gratitude, and psychological capital can comprehensively explain the academic performance of college students. This result not only states the importance of the parent-child relationship as a family environment factor for college students’ gratitude, psychological capital, and academic success. It highlights gratitude and psychological capital as more critical individual internal factors, leading to more positive academic performance.

## Research Limits

This study had certain limitations. First, we only investigated the academic performance of college students from the Yunnan Province of China. Future studies could attempt to replicate and verify our findings using a more extensive survey. Second, we explored the mediating role of gratitude and psychological capital in the effects of parent–child relationships on academic performance using a cross-sectional study. Longitudinal studies or interviews could be included in future studies. Finally, the parent–child relationship was measured among college students, whereas parent–child interactions should be two-way. In future research, parent–child relationships should be measured among the parents of college students as well, and data analysis should be conducted to obtain more empirical findings.

## Conclusion and Suggestions

Overall, this study constructed a chain-mediating model to verify that the parent–child relationship directly affected the academic performance of college students. Similarly, gratitude and psychological capital exerted a mediating role between parent–child relationships and the academic performance of college students. Furthermore, it was established that gratitude positively predicted psychological capital, and that gratitude and psychological capital had a chain-mediating effect between parent–child relationships and college students’ academic performance. According to the findings, good parent–child relationships positively affect gratitude cultivation, promote psychological capital, and academic success among college students. In comparison to the parent–child relationship as a family environment factor, gratitude and psychological capital are more proximal personal factors influencing college students’ academic performance. As a result, we advise college administrators to take the following measures.

First, colleges and universities should pay more attention to the role of parent–child relationships, which influence an individual’s positive emotional development and academic success, by promoting positive parenting styles in multiple ways and guide parents and students to handle conflicts and contradictions properly. On the one hand, parents should be encouraged to create a more democratic family environment in which college students can freely express their ideas and communicate with their parents. Colleges and universities could encourage parents to listen to their children’s opinions, keep up with the times, improve their ability to accept new things, new dynamics, and new lifestyles, and avoid adherence to old ways and parental authority in order to build an excellent parent–child communication and family atmosphere. On the other hand, psychology-related courses could be introduced to help college students understand and deal with parent–child conflicts positively and improve students’ self-adjustment ability to maintain a more benign parent–child relationship. Students could gain support from their families in this way to cultivate a sense of gratitude, develop psychological capital, and thus improve their academic performance.

Second, colleges and universities should prioritize gratitude education and incorporate it into their educational objectives. Positive development in children and adolescents can be aided by gratitude education. Furthermore, gratitude interventions are included in adolescent positive development programs (Bono and Froh, 2009; Lerner et al., 2009). According to clinical psychologists, effective gratitude interventions include gratitude lists, gratitude contemplation, and behavioral expressions of gratitude (Wood et al., 2010). In order to strengthen the gratitude education of college students and promote them to face life and study positively and achieve good academic performance, colleges and universities can implement gratitude intervention in several ways to guide college students to pay attention to and record good things in their lives and cultivate positive life orientations; enhance their reflection on helping nature, society, and others, and cultivate the understanding of gratefulness, return and cherish; to advocate them to regularly take part in social welfare activities and express gratitude by doing what they can.

Third, colleges and universities should focus on improving students’ psychological capital. Psychological capital is an essential internal motivation that can quickly and effectively improve an individual’s academic performance. In daily life and study, several methods could be adopted to enhance the psychological capital of college students and cultivate a healthy psychological state, such as setting mental health courses with the theme of psychological capital and helping students understand the basic knowledge and recognize its importance and paying attention to self-improvement of psychological capital; encouraging students state their opinions through team activities to cultivate self-confidence and a sense of responsibility; guiding students to face difficulties and stress correctly to cultivate an optimistic attitude toward life.

## Data Availability Statement

The original contributions presented in the study are included in the article, further inquiries can be directed to the corresponding author.

## Ethics Statement

Ethical review and approval was not required for the study on human participants in accordance with the local legislation and institutional requirements. The patients/participants provided their written informed consent to participate in this study. Written informed consent was obtained from the individual(s) for the publication of any potentially identifiable images or data included in this article.

## Author Contributions

JL contributed to the conception of the study and drafted the manuscript. JH served as the research advisor. JL and JH contributed significantly to the data analysis and manuscript preparation. ZH and XZ collected the data and worked as writer’s assistants. All authors contributed to the article and approved the submitted version.

## Conflict of Interest

The authors declare that the research was conducted in the absence of any commercial or financial relationships that could be construed as a potential conflict of interest.

## Publisher’s Note

All claims expressed in this article are solely those of the authors and do not necessarily represent those of their affiliated organizations, or those of the publisher, the editors and the reviewers. Any product that may be evaluated in this article, or claim that may be made by its manufacturer, is not guaranteed or endorsed by the publisher.
